# Actionable pathways: interactive discovery of therapeutic targets using signaling pathway models

**DOI:** 10.1093/nar/gkw369

**Published:** 2016-05-02

**Authors:** Francisco Salavert, Marta R. Hidago, Alicia Amadoz, Cankut Çubuk, Ignacio Medina, Daniel Crespo, Jose Carbonell-Caballero, Joaquín Dopazo

**Affiliations:** 1 Computational Genomics Department, Centro de Investigación Príncipe Felipe (CIPF), Valencia, 46012, Spain; 2 Bioinformatics in Rare Diseases (BiER), Centro de Investigación Biomédica en Red de Enfermedades Raras (CIBERER), Valencia, 46012, Spain; 3 HPC Service, University Information Services, University of Cambridge, Cambridge, CB3 0RB, UK; 4 Functional Genomics Node, (INB, PRB2, ISCIII) at CIPF, Valencia 46012, Spain

## Abstract

The discovery of actionable targets is crucial for targeted therapies and is also a constituent part of the drug discovery process. The success of an intervention over a target depends critically on its contribution, within the complex network of gene interactions, to the cellular processes responsible for disease progression or therapeutic response. Here we present PathAct, a web server that predicts the effect that interventions over genes (inhibitions or activations that simulate knock-outs, drug treatments or over-expressions) can have over signal transmission within signaling pathways and, ultimately, over the cell functionalities triggered by them. PathAct implements an advanced graphical interface that provides a unique interactive working environment in which the suitability of potentially actionable genes, that could eventually become drug targets for personalized or individualized therapies, can be easily tested. The PathAct tool can be found at: http://pathact.babelomics.org.

## INTRODUCTION

Diagnostic strategies are rapidly changing in cancer and other diseases because of the availability of increasingly affordable genomic analysis (1). Therapies that specifically target genetic alterations are probing to be safer and more effective than traditional chemotherapies when used in the adequate patient population (2). Actionable targets with therapeutic potential are discovered through empirical associations between the abundance of specific proteins (or transcripts) or the presence of specific mutations and clinical outcomes, and can be assessed by massive sequencing (3). However, single-gene biomarkers have a limited predictive power and frequently only partially account for the fundamental cellular processes responsible for tumorigenesis or therapeutic response (4). Since signaling pathways play a crucial role in these processes, the analysis of its activity should provide better resolution in the development of biomarkers linked to cellular function. We recently proposed the use of mechanism-based biomarkers derived from models of cell signaling activity that account for disease mechanisms or for drug mechanisms of action (5,6). Such models are based on the analysis of the collective contribution of genes to the final signal transmission across signaling pathways. The individual contributions are deduced from gene expression values (6) but gene mutations can also be easily integrated in the model (7). Actually, different types of mechanism-based biomarkers have recently proven to be superior to conventional biomarkers in predicting complex clinical parameters such as bad prognostic (4) or drug sensitivity (8). Thus, the use of models of signaling networks constitutes a promising strategy for the prediction of disease outcomes or responses to therapeutic interventions.

Here we present PathAct, a web server that assess how interventions over genes (knockouts—KOs, over-expressions or drug treatments) can affect to signaling pathways and, ultimately, to the cell functionalities triggered by them. PathAct implements improved robust models of signaling pathways, taken from KEGG (9) and based on our previous work (5,6), within an advanced graphical interface that provide a unique interactive working environment in which potentially actionable genes, that could eventually become drug targets, can be easily assayed alone or in combinations.

## PROGRAM STRUCTURE

PathAct uses the measurements of gene expression in a given condition (diseased tissue biopsy, cell line, etc.) as reference and calculates the signaling activity of all the signaling circuits represented in the pathways. Then, within the interactive workspace, users can easily make interventions on this reference condition. Interventions consist on gene deactivations (simulating KOs) or gene activations (simulating over-expressions). The consequences of these interventions are calculated and the resulting condition is compared to the reference condition, highlighting the differences.

By default, PathAct is used in anonymous mode, which implies that after the session is finished, the results obtained are lost. Alternatively, users can choose sign up for an account. The functionalities are exactly the same but the all the results are stored in the server and available for future sessions.

### Data upload

**Figure 1.**
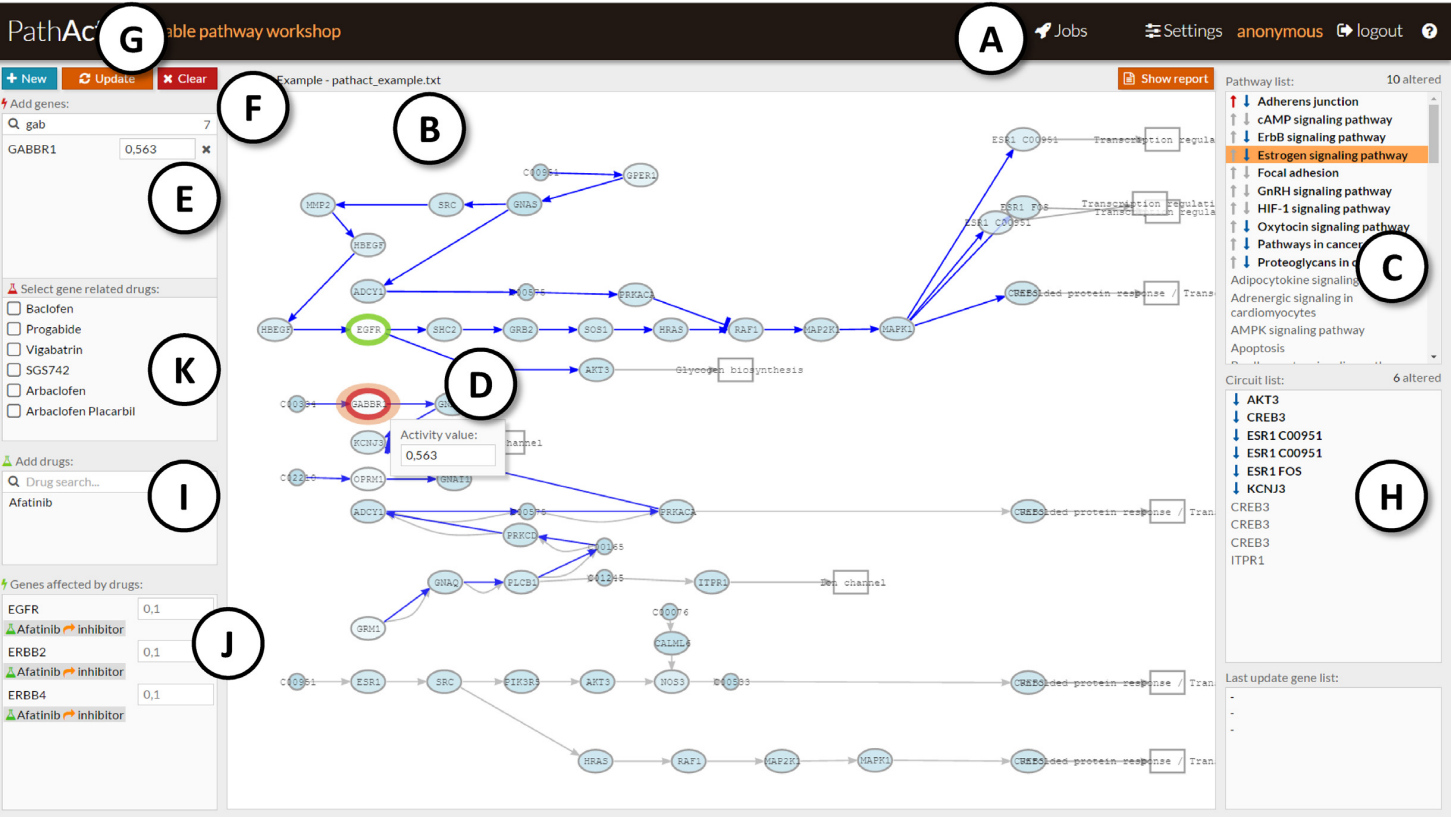
Snapshot of the PathAct working environment. (**A**) *Jobs* panel, to upload data; (**B**) *Pathway viewer* panel, where pathways are displayed; (**C**) *Pathway list* panel, where the pathway to be displayed is selected; (**D**) *Activity value* window, that reports the gene activity value; (**E**) *Add gene* panel, lists the gene selected for the intervention; (**F**) *Add gene* box, where genes can be typed; (**G**) *Update* button, simulates the intervention; (**H**) *Circuit List* panel, contains the circuits in the selected pathway; (**I**) *Add drugs* panel displays drugs typed in the Add drugs box; (**J**) the *Genes affected by drugs* panel contain the genes targeted by the selected drugs; (**K**) *Select gene related drug* list panel contains drugs known to target genes selected in the *Add genes* list.

PathAct uses normalized gene expression values that can be uploaded through the *Jobs* panel (Figure 1A). The input file format is a simple text file with two columns: standard gene identifier (Ensemble gene, gene name, Entrez ID and the most common microarray probe identifiers) and normalized expression value. If probe expressions are provided, the corresponding gene expression values are calculated as the mean value of all the probes mapping in each gene. If more columns with additional samples are provided, the user has to choose the sample to analyze within the input file window. The data file can easily be exported from the typical excel file in which results of microarray or RNA-seq experiments are provided to the users.

Since only one sample at a time is analyzed, normalization, that typically requires of several samples, must be carried out previously by external programs such as the Babelomics (10) or others.

### Gene interventions

Once the file corresponding to the reference condition is uploaded, PathAct calculates the activity status of all the signaling circuits in which the 58 KEGG pathways analyzed can be decomposed. Here, we focus on effector proteins, at the end of the pathways, which are the ultimate responsible for the cellular response to stimulus by triggering specific cell functionalities. Therefore, a signaling circuit is defined here as the sequence of proteins that connect an effector protein back to all the possible receptor proteins from which the signal transmission is initiated upon stimulus reception (5,6). PathAct uses a simplified version of the probabilistic models previously reported (5,6) in which the normalized gene expression values are directly taken as proxies of gene activity (instead of using empirical distributions as in the original formulation of the method), which expands the use of the method to other technologies beyond Affymetrix microarrays, such as RNA-seq. Then, the signal is propagated from the receptor nodes to the final effector nodes in a similar way that in the probabilistic model, as a product of probabilities of gene activity, considering that both activations and inhibitions can coexist within circuits. The circuits, colored according to their activity status, are represented within the pathways in the graphical working environment (*Pathway viewer* panel, Figure 1B). The *Pathway list* panel (Figure 1C) contains the list of pathways. Clicking on them will bring the corresponding pathway to the *Pathway viewer*.

The intervention on a particular gene can be directly carried out within the working environment by setting the cursor over the node targeted, which immediately prompts the normalized value of gene expression (see Figure 1D, *Activity value window*). Such value constitutes its contribution to the signal transmission that could be very relevant if it is a bottleneck for the signal or irrelevant if, for example, lies in one branch of a redundant bifurcation. In addition, the gene can be present in more than one pathway which means that it can be contributing to several signaling circuits simultaneously. The value prompted can be modified to simulate interventions. The selected genes appear in the *Add genes* panel (Figure 1E), where the value of the intervention can be defined. Thus, a KO can be simulated by setting a gene contribution to 0 (or to a low value). Conversely, an over-expression of an inactive gene can be simulated by setting its contribution value to 1 (or to a high value). Additionally, a gene name can be typed in the *Add genes* search box (Figure 1F). Once all the desired interventions have been made, the button *Update* (Figure 1G) recalculates the predicted signaling status of the resulting simulated condition. Then, the simulated condition is compared to the reference condition and circuits are colored according to the activity status changes (red indicates a significant increase in the signaling status and blue a significant decrease). The significance of the change, in absence of a conventional testing scenario, is given by a user-defined threshold, which is set to 2 by default. The *Pathway list* panel summarizes result of the comparison by highlighting pathways in which one or more signaling circuits have significantly changed. The *Circuit List* panel (Figure 1H) displays the significant circuits found within the pathway selected. Clicking on the circuits will interactively highlight them in the *Pathway viewer* panel.

### Simulating drug effects

Also the effect of drugs with known targets (as described in DrugBank) over the different signaling pathways can be studied. The *Add drugs* search box (Figure 1I) allows selecting drugs for simulating the effect on the uploaded system. The genes targeted by the selected drugs appear in the *Genes affected by drugs* panel (Figure 1J). Additionally, any time a gene is selected the *Select gene related drug* list panel (Figure 1K) displays all the drugs known to target such gene. When a drug is selected its known targets are displayed in the *Genes affected by drugs* panel (Figure 1J). If the *Update* button (Figure 1G) is pressed then the effect of the drug over the corresponding gene targets in the *Genes affected by drugs* panel is simulated. Contrarily to the case of gene KOs or over-expressions, in the case of drug action, the predicted gene activity is obtained by multiplying the *drug action weight* by the original normalized target gene expression value. Thus a weight of 0 will set the gene activity to 0 but a coefficient of 0.1 will turn the original gene activity to its 10% (not to a gene activity value of 0.1). Since gene activity values must range between 0 and 1, activity values over 1 after the application of an agonist weight are trimmed to 1. The intensity of the effect produced by the drugs can be modulated in the *Configure Drug Action Weights* window invoked using the *Settings* option of the main menu.

Since many genes participate in more than one pathway and many drugs affect to more than one gene but, simultaneously, signaling circuits are wired with a substantial level of redundancy, the predicted results of drug effects are often unexpected. This fact highlights the importance of comprehensive holistic simulation approaches like the one presented here.

Obviously, unknown off-target effects cannot be predicted. However, they could eventually be inferred by comparing the predicted condition to the real condition measured upon the application of the drug.

**Figure 2.**
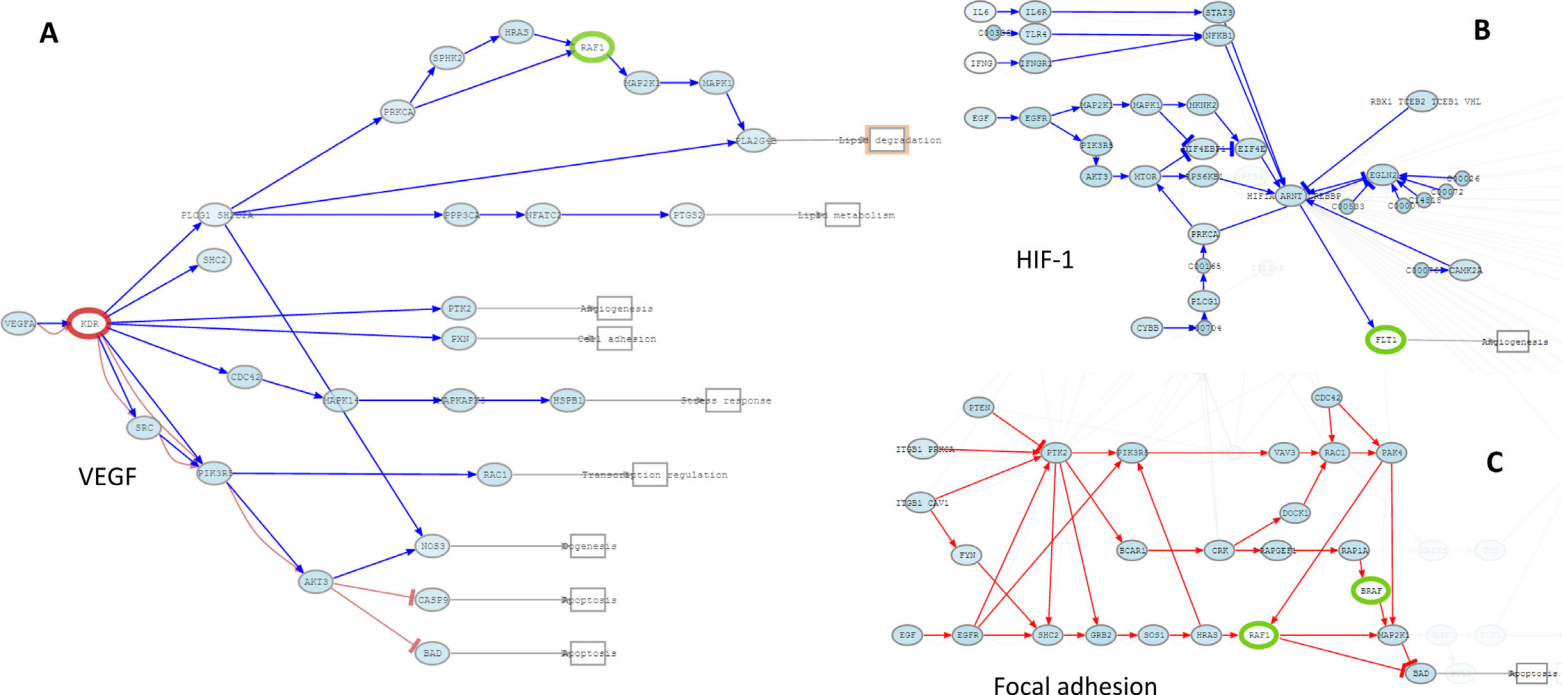
Some pathways affected by Sorafenib. (**A**) VEGF signaling pathway with Angiogenesis inhibited and Apoptosis activated. (**B**) *FLT1* circuit of the HIF-1 signaling pathway with Angiogenesis inhibited. (**C**) *BAD* circuit of the Focal adhesion pathway with Apoptosis activated.

An example of the simulation of the activity of a drug, Sorafenib, is illustrated in Figure 2. Sorafenib is known to inhibit tumor growth by a dual mechanism that involves either a direct effect on the tumor by inhibiting the proliferation in several signaling pathways and/or on an indirect effect, preventing tumor angiogenesis by means of the inhibition of VEGF and other signaling pathways (11,12) (see also FDA prescription information: http://www.accessdata.fda.gov/drugsatfda_docs/label/2013/021923s016lbl.pdf). We use the data in the program example, taken from the TCGA (ID: TCGA.BH.A1FM.11B.23R.A13Q.07), which corresponds to a woman with breast cancer, aged 44, that died 1388 days after diagnostic. Once the data are ready, we choose Sorafenif in the *Add drugs* search box (Figure 1I), which includes nine genes affected: *BRAF, RAF1, FLT3, FLT4, PDGFRB, KIT, FGFR1, RET* and *FLT1*. VEGF signaling pathway, one the canonical angiogenesis pathways, results with most of their circuits down-activated, with Angiogenesis or Cell adhesion functions directly inhibited (Figure 2A). Moreover, the inhibition of *AKT3*, which is an inhibitor of *CASP9* and *BAD*, produces an activation of Apoptosis, thus completing the antitumoral effect of the drug. In addition to VEGF signaling pathway other pathways like HIF-1 signaling pathway (by inhibiting *FLT1* circuit; see Figure 2B) is affected and Angiogenesis is again inhibited. In the Focal adhesion pathway the circuit that triggers Apoptosis undergoes a net activation (Figure 2C). Sorafenib also binds to different MAPK proteins, limiting the tumor growth and inducing apoptosis. Some MAPK are found in different pathways, like RAP-1 or Sphingolipid signaling pathways, and their inhibition provokes inhibition of cell cycle function (see more detail in the worked example 4 in the PathAct documentation). Thus, the prediction of the drug effect fits remarkably well with the description of the effect of the drug in patients and xenografts (11,12).

Combinations of drugs and gene KOs or over-expressions can also be simulated. Therefore, when the Update button is pressed, the corresponding interventions on genes in *Add genes* panels and KOs over the genes in the *Genes affected by drugs* panel are simulated.

### Technical details

PathAct client has been implemented in JavaScript using the HTML5 and SVG standards, which provide a rich and user-friendly interface built with Polymer web components (https://www.polymer-project.org/1.0/) and using CellMaps (http://cellmaps.babelomics.org) functionalities. The backend, which makes the calculations of pathway activities, is written in R. Drugs that target the selected genes are taken from DrugBank (13) through the CellBase (14) webservices. Pathways are taken from KEGG (9).

## DISCUSSION

PathAct has been designed to provide a comprehensive summary of the whole (and often unexpected) effect that interventions on one or several genes have over all the signaling circuits in all the pathways. Moreover, since signals received by receptor proteins trigger functions mediated by the effector proteins at the end of the signaling circuits, both the direct and the long-distance functional consequences of interventions over the genes of the circuit can be straightforwardly revealed within this actionable pathway scenario. PathAct actively contributes to save an enormous amount of time and resources in trial-and-error experiments by allowing highly focused testing of hypothesis of intervention over a reduced number of signaling circuits. In this way this tool will decisively contribute to the acceleration of the discovery of new drug targets and can enormously facilitate application of targeted therapies in personalized treatments. To our knowledge, there are no other tools available that offer a similar functionality.
